# Working Capital Management, Corporate Performance, and Strategic Choices of the Wholesale and Retail Industry in China

**DOI:** 10.1155/2014/953945

**Published:** 2014-07-08

**Authors:** Chuan-guo Li, Hui-min Dong, Shou Chen, Yan Yang

**Affiliations:** Business School of Hunan University, Changsha 410082, China

## Abstract

We examine the influence of strategic choice on working capital configurations and observe how the relationship between working capital ratio and operational performance differs depending on strategy. By clustering the strategic factors of the wholesale and retail industry, we find three categories of strategies: terminal market strategy, middle market strategy, and hybrid strategy. Using the panel data of the listed companies of the wholesale and retail industry as our sample, we analyze the differences in the ways companies configure working capital, the speed with which working capital adjusts to its target, and the effects of working capital on performance for companies that make different strategic choices. The empirical results suggest that working capital is configured and adjusted to its target in different ways under different competitive strategic choices. This effect is finally transferred to influence the relationship between working capital configuration and operational performance.

## 1. Introduction

Working capital and strategic choices are two concepts that have been widely discussed because they impact many aspects of business and financial management. Since Smith [1], working capital has been discussed in holistic terms. The current assets, current liabilities, cash flow, and working capital policy derived from working capital have been examined primarily for their impact on a firm's value [2–5]. Studies on working capital management since Frecka fall into three competing views. Under one view, higher working capital levels allow firms to increase their sales and obtain greater discounts for early payments and, hence, may increase firms' value [6]. Working capital management plays a significant role in the better performance of manufacturing firms [7]. In this line, authors such as Kim et al. suggest that working capital decisions affect firm performance significantly and find that firms with higher values hold a significantly higher investment in working capital than firms with lower values [8–10]. The second view suggests that firms with higher working capital levels may face additional financing expenses which increase their probability of going bankrupt [11]. Firms characterized by high working capital display high sensitivities of investment in working capital to cash flow and low sensitivities of investment in fixed capital to cash flow [12]. From this view, authors such as Shin et al. argue that the firms with higher profits are not motivated to manage working capital and firm performance [13, 14]. Their findings suggest that there is a negative relationship between working capital and firm performance.

Unlike previous studies, other authors argue that the relationship between working capital management and corporate performance is nonlinear [15]. Furthermore, Baños-Caballero et al. find that there is an inverted U-shaped relation between investment in working capital and firm performance [16, 17], which implies the existence of an optimal level of investment in working capital that balances costs and benefits and maximizes a firm's value.

At the same time, the literature on strategy management theory often suggests that a firm's overall performance may be contingent upon the nature of the strategic choices a firm makes [18–21], and the literature on resource-based theory suggests that a firm's strategic choices may be made based on superior resources and may represent the means by which resources are allocated [22–28]. That is, a firm's strategic choice must consider its resource allocation, and different strategic choices and/or resource allocations may lead to different performances. Working capital configuration, an important aspect of company resources, may be affected by the strategic choices, as well. Using the Cobb-Douglas model, Hamlin and Heathfield deduced a relational model of working capital and strategy [3]. They argued that a competitive strategy influences production flexibility and working capital management, and companies dedicated to maintaining competitive advantages should strengthen their working capital management accordingly. Nath et al. suggest that an enterprise's marketing capability, operations capability, and diversification strategy have an integral influence on firm performance [29]. Therefore, the question is will the working capital configuration change with the transformation of strategic choices? If it does, will this relationship affect performance? The prior empirical evidence is largely limited to the relationships between strategic choices and performance [30–32] or between working capital and performance [4, 13]. Thus the impact of strategic choices on working capital and performance has rarely been examined directly. Therefore, it is important to investigate the influence of strategy on working capital configuration and the working capital-performance relationship.

In the present study, the relationship among working capital configuration, performance, and strategic choices is analyzed. The study was conducted in the context of research on strategic choices and working capital configuration (how do strategic choices influence working capital allocation), which has attempted to explain the effects of strategic choices on working capital. Therefore, the research proceeds from the perspective that strategic choices play a deciding role in the influence of working capital on performance.

In the present study, we choose the Chinese wholesale and retail industry as the research objects for two reasons. First, statistics show that the average proportion of current assets to total assets in the wholesale and retail industry is approximately 50% and that of current liabilities to total debt ratio is more than 90% (the data are derived from the Chinese national statistics bureau). The wholesale and retail industry, a bridge connecting producers to customers, pays more attention to working capital than any other industry. Second, the Chinese wholesale and retail industry had been integrating since the late 1980s and growing rapidly after undergoing expansion and adjustment in the early and mid-1990s. The competitive strategy and business pattern of Chinese wholesale and retail enterprises began to diversify with the emergence of a new retail business pattern and new wholesale agents. In addition, the Chinese economy is developing rapidly, with a Gross Domestic Product (GDP) growth rate of approximately 7% annually. The impact of strategic choices on working capital and performance in a rapid development industry in a rapid development country needs to be explored.

Based on the diverse arguments of the above-mentioned studies and to explore the role of strategic choices in working capital and performance in a rapid development industry, we argue that working capital and its capacity to drive operational performance are not always consistent for companies employing different strategies. Specifically, this study seeks to answer the following two empirical questions. First, do firms that make different strategic choices have different working capital configurations, especially when making the adjustment from current working capital to target working capital? Second, if firms with different strategic choices make different working capital decisions, will this finally transfer to business performance in the Chinese wholesale and retail industry?

As the first step of our investigation, we must understand the category of strategy that a company adopts. Describing a strategy with the data at our disposal is the first problem we face. To capture strategic characteristics and measure a strategy with financial statement level data, we explored the index to distinguish strategies based on two important strategic value propositions, “efficiency”, and “cost”. By excavating 10 indicators reflecting capital investment efficiency and cost control ability and clustering the sample according to the characteristics of these 10 indictors, we classify the strategies of listed companies in the wholesale and retail industry into three categories. Based on the strategy recognition and the two-stage working capital adjustment model, the adjustment speed of working capital and its influential factors on different strategic choices are analyzed and compared by panel data. Furthermore, the relationship between working capital ratio and operational performance and the marginal influence of working capital on performance are also examined by panel data analysis. To clarify the essential role that strategy plays in working capital and the working capital-performance relationship, we compare the statistic parameters for samples belonging to different strategies. The empirical results suggest that working capital is configured and adjusted to its target in different ways depending on different competitive strategic choices. This effect is finally transferred to influence the relationship between working capital configuration and operational performance. The marginal influence of the working capital ratio on performance is different with different strategies.

This study contributes to the working capital management literature in a number of ways. First, we construct a model of working capital adjustment based on the target adjustment model of the capital-structure or target debt ratio (leverage) [33] and conduct an empirical study on the adjustment path of working capital under different strategies. Second, the paper investigates the relationship between investment in working capital and firm performance according to different strategies of firms and the marginal influence of working capital ratio on performance with different strategies. Third, we estimate the models by using a panel data methodology to eliminate unobservable heterogeneity and use the generalized method of moments (GMM) to address possible endogeneity problems.

## 2. Hypothesis

### 2.1. Hypothesis Regarding Working Capital Adjustment

There is a specific working capital level which objectively creates enterprise value maximization. Previous work has verified that there is a target working capital level. For example, Baños-Caballero et al. proved that a target cash flow cycle exists in enterprises [34]. If there is a shortage of working capital, enterprises will probably borrow money at a high interest rate at the wrong time to maintain regular operations and credit, thus affecting the ability to pay interests and dividends. However, a high working capital level means there is a substantial amount of liquidity that does not create more economic benefits, which implies that enterprises may lack investment opportunities and potential development will be influenced. Enterprises should maintain a proper working capital level.

Therefore, enterprises need to adjust the holdings and composition of working capital to adapt to market needs. Due to the differences in adjustment costs (such as interest, rent, and conversion cost) and different maintenance costs (if they did not plan to do anything), enterprises' working capital adjustments are different in different strategic types. The clients of enterprises with a terminal market strategy are a single person whose purchasing behavior is at will. Enterprises cannot actually forecast the person, place, time, and product categories of purchase behavior. They may pursue differences, spend more money on temporary marketing outlays to meet customers' needs, and keep more short-term loans to maintain differentiation. Therefore, they may not be concerned much with the adjustment of working capital. However, enterprises with a middle market strategy tend to have fewer monetary funds to reduce opportunity costs and reduce external financing costs to avoid interest. The clients of companies with a hybrid strategy are diverse, meaning that they may be a person or a company. The business of these companies is complex, and therefore they may pay more attention to liquidity and working capital policy and adjust it as soon as possible.

*H1*. Companies with a hybrid strategy make the adjustment from a current working capital ratio to a target working capital ratio the fastest, while companies with a terminal market strategy are slowest and companies with a middle market strategy are in the middle.

### 2.2. Hypothesis Regarding the Working Capital Influence on Performance

We define working capital ratio as (current assets-current liabilities)/current assets. This index reflects not only short-term debt paying ability but also the financial strategy of a company. When working capital ratio falls within a reasonable range, the larger the working capital ratio is, the more long-term the capital is invested in current assets. That is, the more conservative the financial policy that the company employs, the less the financing risk is undertaken by the company. Thus the company has more stable capital as to guarantee the continuity of business operations and, in turn, to safeguard the stable profit of the company. Therefore, we propose hypothesis 2A.

*H2A*. The link between working capital ratio and performance is positive.

Because the strategic objectives of different strategies are different, the internal resource allocation scheme, cost control techniques, and differentiation extent are different for companies that make different strategic choices, including the working capital configuration. Given that many scholars have demonstrated that either working capital or strategy will influence performance and we have discussed above how strategic choices will affect working capital management, we hold that performance will differ based on different working capital management plans and strategic choices. In this case, the marginal influence of working capital ratio on performance will be different with different strategies.

*H2B*. The marginal influence of working capital ratio on performance will be different with different strategies.

## 3. Data and Variables

### 3.1. Data Sources

In this paper we demonstrate through empirical analysis the influence of strategic choices on working capital management and the way that working capital affects performance. This analysis requires three types of data: indicators in the depiction of strategy, indexes describing working capital configuration and performance, and data for control variables. For this reason, our sample covers financial data in the annual financial statements from 2008 to 2012 of 113 Chinese listed companies in the wholesale and retail industry. All of the data in this paper are collected from the China Stock Market & Accounting Research Database (CSMAR) (GTA Information Technology Co., Ltd., Shenzhen, China) and TinySoft (TinySoft Corp., Shenzhen, China) in China. The selection criteria of our sample are as follows. First, firms that are B-shares enterprises and oversea-listed companies are excluded. Second, we choose data concerning the listed companies in the wholesale and retail industry from 2008 to 2012 as the research object because the data from this time period is the most complete and development is relatively fast in this time window. Third, we eliminate sample items with data missing from one or more than one years to ensure the data integrity. A total of 475 observations of 95 listed companies in the Chinese wholesale and retail industry from 2008 to 2012 remained.

### 3.2. Variables

#### 3.2.1. Strategy Variables

Porter identifies two generic ways in which a firm can gain a sustainable competitive advantage: cost leadership and differentiation [35]. The wholesale and retail industry is no exception. To obtain competitive advantage, wholesale and retail enterprises that pursue low costs pay more attention to cost and expense control, the assets turnover cycle, and capital use efficiency. Companies that pursue differentiations focus on products, sales, and service. Therefore, we select indicators of capital investment efficiency, cost control, and development ability.

*Capital Investment Efficiency*. Capital investment has been considered a key indicator of strategic evaluation. Based on capital investment status (plant, equipment, current assets, etc.), we can judge capital quality, operating efficiency, management level, and cost control capacity [36]. Therefore, the ratio of fixed assets to profit, fixed asset turnover ratio (sales to net fixed assets), and current asset turnover ratio are used to measure capital investment efficiency.

*Cost Control*. Enterprises with a cost leadership competitive advantage have a strong motivation to control cost and improve operational efficiency. They strive to lessen costs by controlling different types of expenses, the financial cost of external financing, the selling cost for marketing, and the administrative cost for day-to-day administration. Conversely, enterprises that pursue differentiations always give priority to marketing capability cultivation. They emphasize the importance of advertisements, service, brand, and so forth, and they are most likely to finance through debt and stock when necessary [36, 37]. In addition, enterprises may ignore financial expenses control and spend more on advertisements and distribution channels in pursuit of differentiations. Thus, the ratio of management expenses to profit, the ratio of financial expenses to profit, the ratio of sales expenses to sales, and the ratio of costs to income are selected to reflect how much enterprises spend on cost control or differentiation.

*Development Capability*. Enterprises committing themselves to differentiation usually tend to invest capital in fixed assets to enlarge their business scope and market share. They sell different products to different target customers by swapping and recombining a variety of resources [38]. Thus, they can easily acquire brand loyalty and gain more profit compared to competitors. Conversely, enterprises devoted to lessening costs always invest less in fixed assets. Therefore, the relative gross margin, the growth rate of fixed assets, and the growth rate of sales proceed are used to reflect development capability.

#### 3.2.2. Measures of Firm Size

Firm size plays an important role in strategy in the wholesale and retail industry. For example, large firms tend to improve their bargaining ability using size as a chip and enjoy various preferential supply policies to achieve economies of scale. Because firm size is not suitable to be used as an indicator to represent strategy, we describe strategic types with firm size and strategic indicators. Firm size is measured by the natural logarithm of total assets.

#### 3.2.3. Measures of Working Capital

Generally, working capital refers to the difference between current assets and current liabilities. This concept implies the financial strategy that a company observes. If working capital is less than 0, the current liabilities are larger than current assets. In this case, the company advocates a radical financial strategy. Otherwise, if the current assets are much larger than current liabilities, that is, part of long-term capital is invested in short-term assets, then the company employs a conservative financial strategy. From this point of view, the larger the working capital is, the more radical the financial strategy is observed and the safer the short-term liabilities are. Therefore, we adopt this definition of working capital in this study. However, to control for the impact of size, we use the proportion of difference between current assets and current liabilities to current assets to represent working capital, namely, the working capital ratio, designated as working capital.

#### 3.2.4. Measures of Working Capital Influencing Factors

*Management Efficiency of Working Capital*. Enterprises with a large-scale inventory and high efficiency in accounts receivable management apparently invest less in current assets to achieve the same growth rate of sales. Conversely, enterprises with a small-scale inventory and low efficiency in accounts receivable management have more working capital to achieve an objective growth rate of sales. Therefore, we select the inventory turnover ratio and accounts receivable turnover ratio to measure working capital efficiency.

*Growth Opportunities*. In general, if operations' management efficiency remains the same, working capital size will increase with the sales growth. However, the relationship between growth opportunities and working capital is controversial. On the one hand, sales growth leads to the growth of accounts receivable and inventory. On the other hand, enterprises with better performance will easily attract outside investment and therefore do not need much more cash and short-term loans that can be invested in other plans to gain more profit. Growth rate of sales proceed is used to measure growth opportunities.

*Operational Cash Flow*. Enterprises would be willing to increase their current working capital in the short term if they expected that they would have more development opportunities and future cash flow [39–43]. The more operating cash flow the enterprises have, the higher their working capital management level is. Therefore, enterprises' working capital and debt will remain at a low level [44]. Operating cash flow to total assets is used to measure cash flow to eliminate the influence of firm size.

*Fixed Assets Ratio*. The increase in structural assets investment, such as fixed assets, intangible assets, and long-term investments, will lead to the reduction of working capital. Therefore, the fixed assets ratio will affect working capital. The ratio of fixed assets to total assets is used.

#### 3.2.5. Measure of Performance

There are many variables that can reflect performance, such as revenue, capital usage ratio, and return on assets (ROA). However, revenue and capital usage ratio can only reflect business performance to an extent, while ROA can comprehensively reflect business performance [45]. We define ROA as earnings before interest and taxes (EBIT) relative to total assets.

#### 3.2.6. Control Variables

Industry development will influence companies' business performance, so we use the steady of industry demand to control the change of the whole industry. Industry demand uncertainty captures the volatility of industrial demands and is measured by the standard deviation of the industrial average net sales from 2008 to 2012. Many studies have demonstrated that initial performance will affect current performance, and thus we add the steadiness of industry demand and initial performance of the model to control the influences of industry and firm and improve the accuracy of the model to study the marginal influence of working capital on performance for different strategies.

#### 3.2.7. Measures of Working Capital Structure and Efficiency

In the wholesale and retail industry, companies place great value on working capital management to achieve a high turnover rate of current assets. As a result, working capital forms a very important strategic resource influencing strategic choice and is, in turn, constrained by strategy. To investigate the differences in working capital structure and the operational efficiency of working capital under different strategic choices, we decompose current assets into cash, inventory, and receivables and decompose current liabilities into short-term financial assets in terms of the working capital structure. In addition, we use the inventory turnover ratio and accounts receivable turnover ratio to reflect working capital efficiency.

All of the variable definitions are reported in Table 1.

## 4. The Classification and Identification of Strategy

Although enterprises have different types of advantages and disadvantages in relation to their competitors, the two most basic competitive advantages that form the fundamentals of competitive strategy are low cost and differentiation [35]. Researchers commonly measure enterprises' strategies by considering their abilities to keep costs low and products differentiate. In the wholesale and retail industry, low cost or cost control is even more appreciated given the nature of the industry.

We classified strategies in the wholesale and retail industry by clustering the sample of strategic factors. All of the data used in this paper are on financial level data because they permit an explicit gauge for measuring “realized strategies” rather than “intended strategies” [46]. In addition, by using financial statement level data, these measures are not prone to the perceptual biases noted in the strategy literature [47]. With two independent factors obtained from the factor analysis, a hierarchical cluster analysis was conducted with the goal of verifying whether there were differences between groups of firms and then determining the optimal cluster number and types of strategy.

Table 2 reports the factor analysis results. As the value we obtain from the Kaiser-Meyer-Olkin test (KMO) is 0.6886, the *P* value of the Bartlett test is 0.000, and the overall contribution is 96.54%; the sample is suitable for factor analysis and consistent with our expectations. The results indicate that there are two factors. The first factor (eigen value is 2.44) comprises three indicators, namely, the ratio of fixed assets to profit, the ratio of management expenses to profit, and the ratio of financial expenses to profit. Because all of these indicators reflecting this factor are related to the input-output relationship, we designate the factor input-output efficiency. The higher (lower) the value is, the lower (higher) the input-output efficiency is. The second factor (eigen value is 2.38) is comprised of four indicators, that is, the ratio of sales expenses to sales, the relative gross margin, the ratio of costs to income, and the fixed assets turnover ratio and the current assets turnover ratio. All of these indicators reflect capital investment and efficiency, and thus we name it capital investment efficiency. The higher (lower) the value, the higher (lower) the companies' cost consumption and assets turnover ratio. In conclusion, the higher (lower) the factor scores, the more likely the firms' expenditures on capital are large (small). In this case, all of the expenses of enterprises are high, which can reflect that companies seek different and expanded markets based on their own ability. Conversely, the lower the factor scores are, the more likely the enterprises spend less on capital and control costs strictly.

Based on the two factors obtained from the factor analysis, we create a strategic classification through hierarchical clustering and *K*-mean value clustering and classify strategies into three classes according to the characteristics of the two factors, as shown in Table 3. Furthermore, an analysis of variance (ANOVA) is adopted to examine the difference in these two factors for companies operating different strategies.

Firms in cluster 3 are high in input-output efficiency, capital investment efficiency, and firm size, with values of 0.29, 0.38, and 21.67, respectively. With these characteristics, we hold that enterprises in this class may pay more attention to brand promotion, marketing, product design, and characteristics improvement, and they may thus dedicate themselves to shortening the cycle of new product development, increasing product categories, and improving product packaging. These characteristics coincide with wholesale companies. In addition, the ratio of wholesale companies is 73.20%, so we designate it the middle market strategy.

As shown in Table 3, firms in cluster 2 have a low level of input-output efficiency, capital investment efficiency, and firm size, with values of −0.32, −1.29, and 21.28, respectively. Thus, we hold that enterprises in this class may advocate cost controlling, economies of scale, and using the advantage of value chain to realize trading internalization and to maximally reduce purchasing expenses. They try to carry out market segmentation to increase product sales, extend market share, develop new markets, spread market risk, and expand advantages. These characteristics are similar to retail companies. Furthermore, the ratio of retail companies is 75.23%, so we name this the terminal market strategy.

The values of input-output efficiency, capital investment efficiency, and firm size of enterprises in cluster 3 are in the middle of the three classes. We claim that enterprises in this class pay attention to cost control, brand promotion, and new production development at the same time. However, they primarily achieve a reasonable balance between cost control and differentiation and do not excessively emphasize either side. Their capital investment efficiency is relatively moderate and reasonable. Companies in this class have both wholesale and retail businesses, and the ratio of these companies is 82.72%. Therefore, we name the third strategic type the hybrid strategy.

## 5. The Working Capital Configuration in Different Strategic Choices

### 5.1. Working Capital Structure and Operational Efficiency under Different Strategic Choices

The ANOVA results are presented in Table 4.

The mean value of the working capital ratio in the three strategic choices is different. It is the highest in the middle market strategy and the lowest in the terminal market strategy.

In the middle market strategy, the average inventory turnover rate is 27.9, and the average accounts receivable turnover ratio is 41.85. However, in the terminal market strategy, the average inventory turnover rate is 13.99, and the average accounts receivable turnover ratio is −3.11. Based on the results shown above, we can conclude that working capital management efficiency in the middle market strategy is the highest, while that in the terminal market strategy is the lowest.

In the middle market strategy, enterprises' average ratios of receivables and inventories to current assets are 16.77 and 30.81, respectively, which are higher than those in the terminal market strategy and the industry average. Meanwhile, the average ratios of cash to current assets and short-term loans to current liabilities are 28.23 and 29.46, respectively, which are lower than the industry average and those in the terminal market strategy. The ratios of cash, inventories, receivables to current assets, and short-term loans to current liabilities in the hybrid strategy are in the middle. Therefore, we can conclude that the efficiency and structure of working capital are different with different strategies.

Because the credit policy of companies in the middle market strategy is relatively easy and the size of investment in working capital is high, the working capital efficiency and the ratios of receivables and inventories to current assets in the middle market strategy are the largest, while the credit policy of companies in the terminal market strategy is relatively strict and the cash holdings are large, meaning that they have a high short-term debt paying ability and thus usually keep relatively high short-term borrowing to lower financing costs and opportunity costs. Therefore, the ratio of cash to current assets and the ratio of short-term loans to current liabilities in the terminal market strategy is the largest.

The results above verified the validity in classifying firms into three types and proved that working capital management is different with different strategic choices. To assess whether strategic choices influence working capital management, we analyze the working capital adjustment speeds of the different strategic choices in the following section.

### 5.2. The Difference in Working Capital Adjustment Speed and Its Influential Factors in Different Strategic Choices

#### 5.2.1. Model of Working Capital Adjustment

Many scholars have proven that there is a target working capital rate in a company. In an ideal condition, the current working capital level should be equal to the ideal value. However, because of the adjustment cost, the actual working capital level will not be fully equal to the ideal value. Because companies need to possess a certain amount of cash, accounts receivable and inventory due to the instability of business management, current assets, and current liabilities, the composition of working capital is constantly being replaced. Based on the target adjustment model of the capital-structure or target debt ratio (leverage) [33], the adjustment model on working capital is as follows:

(1) $$\begin{array}{c} {\text{WC}}_{i,t}=\left(1-\alpha \right){\text{WC}}_{i,t-1}+\alpha \beta_{0}+\alpha \overset{n}{\underset{j=1}{\sum }}\beta_{j}X_{i,t,j}+\mu_{i,t}+d_{t}+v_{i,t}, \end{array}$$

where WC_*i*,*t*_ is the working capital level of firm *i* at time *t*, *X*_*i*,*t*,*j*_ is a set of *j* working capital level determinants of firm *i* at time *t*, including company's operating condition, cash flow and working capital management efficiency, and company asset allocation, and *μ*_*i*,*t*_ is the error term. The target-adjustment coefficient *α* measures the relevance of the transaction costs and is assumed to be a samplewide constant, which represents adjustment degree. The error term in our models has been split into three components: first, the individual or firm-specific effect *μ*_*i*,*t*_, second, *d*_*t*_, which measures the time-specific effect by the year dummies, and finally, *v*_*i*,*t*_, which is the random disturbance.

#### 5.2.2. Analysis of Working Capital Adjustment

Using Model 1 and the sample of listed companies in the wholesale and retail industry from 2008 to 2012 specified above, and by GMM estimation of the panel data, we obtain the results shown in Table 5.

The GMM results show that there is no second-order serial correlation and it is valid to use time dummy variables. The previous year's working capital ratio has a positive influence on the current working capital level, and the relationship is significant at the one-percent level. The size of the coefficient of the lagged working capital level variable (1 − *α*), as specified in Table 5, was in the range of 0.267 to 0.500 for the sample as a whole. Accordingly, the parameter *α*, which measures the adjustment speed of the current working capital ratio towards a target working capital ratio, distributes over the range of [0.500, 0.733]. Thus working capital adjusts to a quite normal degree for the adjustment cost in the wholesale and retail industry. However, disparities in adjustment speed are found to be significant among the samples of firms with different strategic choices though the adjustment is in a normal range. On average, firms operating the hybrid strategy enjoy the highest adjustment speed, while those in the terminal market strategy adjust their working capital in the lowest speed.

As argued by Ozkan [48], the adjustment decision is a trade-off between the adjustment (transaction) cost involved in moving towards a target ratio and the cost of being diverted from optimal ratio. If the latter is greater than the former, then the estimated coefficient (1 − *α*) should be close to zero, and firms will try to adjust their working capital ratio to the target as soon as possible. Based on the estimated adjustment speed, convergence towards a target seems to explain much of the variation in firms' working capital ratios. Specifically, firms adopting the terminal market strategy reported a target-adjustment coefficient that, although statistically significant, is close to 0.5 and, therefore, makes effects of the adjust very small. Instead, firms that adopted the middle market strategy move quicker towards their target working capital than those that adopted the terminal market strategy to maintain working capital and adapt to market changes actively. However, firms with the hybrid strategy adjust their working capital to the equilibrium level the most quickly. Because enterprises' capital investment efficiency is moderate and close to the industry average in the hybrid strategy, they pay more attention to business management, and therefore their working capital ratio is closer to the target working capital ratio and their working capital adjustment speed is the fastest. These results support hypothesis 1.

As previous studies have shown, working capital structure depends on several firm-specific characteristics. The results generally show that the choice of working capital level is a negative function of the inventory turnover ratio and the accounts receivable turnover ratio. When working capital management efficiency is higher, working capital holdings are lower. Of the three strategies, working capital management efficiency has the greatest effect on working capital in the terminal market strategy, and the relationship between the accounts receivable turnover ratio and working capital is not significant.

The link between operating cash flow to total assets and working capital ratio is negative in the terminal market strategy. When operating cash flow grows as a result of business activities, enterprises may have good working capital management ability, and thus firms tend to hold less working capital. However, the link between operating cash flow to total assets and working capital is not significant.

The relationship between fixed assets to total assets and working capital ratio is always negative, and it is slightly stronger for enterprises with the terminal market strategy. When there are more fixed assets, the working capital ratio is lower.

The relationships between the growth rates of sales proceed and working capital ratio are different in the hybrid strategy and the middle market strategy. The positive link between the growth rate of sales proceed and working capital ratio indicates that enterprises have more free money to allocate to working capital and meet the temporary needs of diversification. Conversely, a negative link between the growth rates of sales proceed and working capital ratio is exhibited by the hybrid strategy. Enterprises with this strategy neither overused nor oversaved capital. Therefore, when the growth rate of sales proceed is larger, the enterprises tend to allocate more money to business. The link between the growth rate of sales proceed and working capital ratio is not significant in the terminal market strategy.

## 6. Influence of Working Capital on Performance in Different Strategies

Strategic fit is a core concept in normative models of strategy formulation, and the pursuit of strategic fit has traditionally been viewed as having desirable performance implications [49, 50]. Strategic and organizational theory argues that particular structures are more appropriate for given strategies, and changes in environmental conditions and organizational resource or structures require changes in the choice of strategy. The internal resources must be fitted, integrated with strategy, and could be converted into a competitive advantage [51]. According to the empirical analysis of working capital adjustment, investment in working capital and the ratios of working capital structure and efficiency significantly change under different strategies. Furthermore, the effects on the target working capital ratio are different depending on factors such as working capital management efficiency, operating cash flow, business operations, asset allocation. Based on these findings, we analyze the moderating effects of strategic choice on the relationship between working capital management and corporate performance as well as the marginal effects of working capital on performance under different strategies. In addition, we analyze the fit between the strategic choice and working capital management based on strategic fit theory.

### 6.1. Other Variables 

#### 6.1.1. Measures of Strategic Types

We use dummy variables to reflect strategic types. As we have three types of strategy in the wholesale and retail industry, we design two dummy variables, IO and IS, to represent these three types of strategy in the following way. We use I to reflect the strategy that a company employs, *A* to reflect the hybrid strategy, *B* to reflect the terminal market strategy, and *C* to reflect the middle market strategy:

(2) $$\begin{array}{c} \text{IO}=\{\begin{array}{cc} 1 & I\in A\\ 0 & I\in A,\;\;I\in C \end{array}\\ \text{IS}=\{\begin{array}{cc} 1 & I\in B\\ 0 & I\in A,\;\;I\in C. \end{array} \end{array}$$

If IO = 0 and IS = 0, then the company employs the middle market strategy, which is the reference point for the hybrid strategy and the terminal market strategy.

#### 6.1.2. Measure of Interaction Factors of Marginal Influence

To investigate the differences in the way that working capital affects performance, we design interaction factors of strategy and the working capital ratio by multiplying dummy variable that reflect the strategy and the standardized working capital ratio; namely, IO × WC and IS × WC (Table 6). If IO × WC ≠ 0 but IS × WC = 0, then the sample of companies operating the hybrid strategy is represented. If IO × WC = 0 but IS × WC ≠ 0, then the sample of companies operating the terminal market strategy is represented, and if IO × WC = 0 and IS × WC = 0, then the sample of companies operating the middle market strategy is represented.

#### 6.1.3. Selection of Working Capital Ratio 

**Figure 1:**
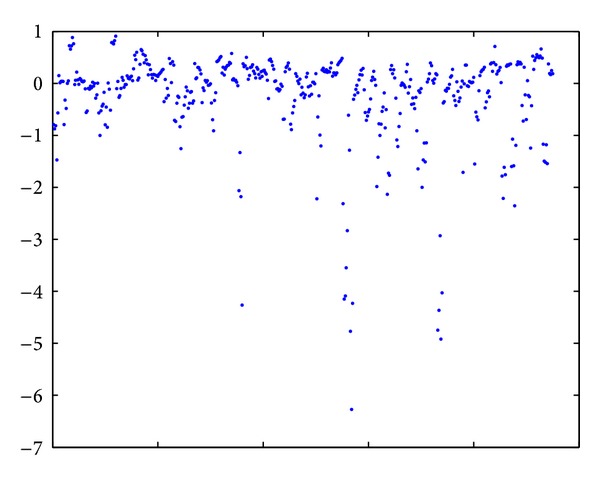
Distribution of working capital in the whole industry.

Table 7 and Figure 1 report the distribution of working capital across the whole industry We can conclude that most of the working capital ratios are between [−2,1] and those between [−6.5, −2] only take 4.4% of the sample. Companies whose working capital ratios are between [−6.5, −2] have substantially more current liabilities than current assets. In other words, the working capital management of these companies is very radical and is not the same as normal working capital management. Thus, we eliminate these risk samples to better reflect the working capital management of most normal companies.

### 6.2. Model

We add control variables to control the influence of factors, with the exception of working capital, that affect firm performance. The interaction terms are also added in Model 2 to discuss the marginal effect of working capital on performance in different strategies:

(3) ROAi,t=α1+γ1WCi,t+γ2IOi,t+γ3ISi,t +γ4IO×WCi,t(1)+γ5IS×WCi,t(2) +γ6IDU+γ7ROAi,t−1+εi,t.

IO × WC_*i*,*t*_^(1)^ reflects the working capital ratio in the current hybrid strategy. IS × WC_*i*,*t*_^(2)^ reflects the working capital ratio in the current terminal market strategy. IDU is the control variable and captures the volatility of industrial demands. ROA_*i*,*t*_ is the dependent variable. *α*_1_ is the intercept. *γ*_1_, *γ*_2_, *γ*_3_, *γ*_4_, *γ*_5_, *γ*_6_, and *γ*_7_ are the coefficients. According to the model and dummy variables, we assert that *γ*_1_ reflects the marginal effect of working capital on performance. *α*_1_ reflects the intercept of performance in the middle market strategy. (*γ*_1_ + *γ*_4_) reflects the marginal effect of working capital on performance. (*α*_1_ + *γ*_2_) reflects the intercept of performance in the hybrid strategy. (*γ*_1_ + *γ*_5_) reflects the marginal effect of working capital on performance. (*α*_1_ + *γ*_3_) reflects the intercept of performance in the terminal market strategy.

### 6.3. Working Capital-Performance Relationship in Different Strategies

Based on Model 2 and GMM estimation of the panel data, the results of the regression analysis of the influence of working capital on performance in different strategies are reported in Table 8. Model 1 included working capital and controls; Model 2 added the effects of the different strategies (IO and IS) and their interactions with working capital (IO × WC_*i*,*t*_^(1)^ and IS × WC_*i*,*t*_^(2)^). Wald Test and R-square for these models indicate significant explanatory power.

In models 1 and 2 in Table 7, the effects of working capital on performance are positive and significant (*γ*_1_ = 0.012, *P* < 0.011 in Model 1; *γ*_1_ = 0.032, *P* < 0.004 in Model 2). These results strongly support hypothesis 2A, which indicated that the level of working capital would have a positive relationship with firm performance.

Hypothesis 2B predicted that the strategic choice would affect the relationship between the level of working capital and firm performance. In Model 2 of Table 8, the interaction of strategic choice IO and working capital is not significant (*γ*_4_ = −0.018, *P* < 0.136), but the interaction of strategic choice IS and working capital is negative and significant (*γ*_5_ = −0.019, *P* < 0.098). These results suggest that the terminal market strategy moderates the effect of the level of working capital on firm performance, as we predicted in hypothesis 2B.

### 6.4. Marginal Effect of Working Capital on Performance in Different Strategies

To further examine the marginal effects of working capital on performance in the different strategies, we first take the partial derivatives of the performance in Model 2 with respect to the working capital ratio. ∂ROA/∂Working Capital reflects the marginal influence of working capital on performance:

(4) $$\begin{array}{c} \frac{\partial \text{ROA}}{\partial \text{Working Capital}}=\gamma_{1}+\gamma_{4}{\text{IO}}_{i,t}+\gamma_{5}{\text{IS}}_{i,t}. \end{array}$$

According to the regression results of models 1 and 2 in Table 7, the marginal effects of working capital on performance in different strategies and in the industry as a whole are shown in Table 9.

Based on Table 9, we can see that the marginal influence in the middle market strategy is the highest, while that in the terminal market strategy is the lowest.

Most companies with the terminal market strategy are retail companies, which are characterized by scattered trading volume, frequent trading times, different sizes of business outlets, wide distribution of business, and so forth. Compared with wholesale companies, the volume of business of these companies is smaller. Therefore, they pay more attention to the increase in capital turnover rate and capital liquidity, and thus the ratio of operating fund to current liabilities is higher. Due to the dependency of working capital in the operating process, the generation of business performance mostly relies on “small profits but quick turnover” and capital liquidity. Therefore, the marginal effect of working capital on performance is very low.

However, companies with the terminal market strategy mostly are wholesale companies, which usually distribute in large cities. These companies differ from retail companies insofar as they have a bigger trading size, lower trading frequency, more rational trade, fewer transaction projects, and so forth. Therefore, they pay less attention to capital liquidity compared with terminal market companies, and their generation of performance mostly relies on trading volume. Thus the marginal effect of working capital on performance is the largest.

Companies with the hybrid strategy operate both wholesale and retail businesses, so the current liability needs and the capital turnover rate are relatively in the middle. Thus, the marginal influence of working capital on performance is in the middle, as well. These results support hypothesis 2B.

**Figure 2:**
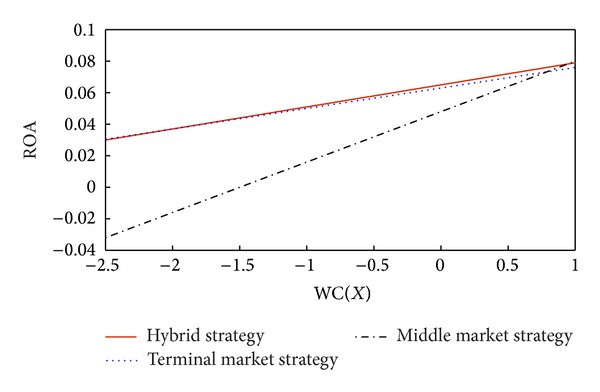
Marginal effects of working capital on performance in current strategies.

According to the intercept and margin calculated above, we draw schematics to analyze the marginal effects as shown in Figure 2. Meanwhile, when we observe the source data of ROA, we find that there are 37 observations that are below 0, while the other 438 observations are above 0.

According to Figure 2, the companies with the middle market strategy should pay more attention to the marginal impact of working capital on firm performance than companies with the terminal market strategy or the hybrid strategy. The level of working capital is higher, the effect of working capital on performance is higher, and company performance will also be better.  Table 10 reports the performances in the different strategies when the working capital ratio is in different intervals.

When the working capital ratio is lower than −1.63, the performance in the terminal market strategy is the best and the performance in middle market strategy is the lowest. When the working capital ratio is between [−1.63, 0.75], the performance in the hybrid strategy is the best and the performance in middle market strategy is the lowest. When the working capital ratio is between [0.75, 0.95], the performance in the hybrid strategy is the best and the performance in the terminal market strategy is the lowest. When the working capital ratio is higher than 0.95, the performance in the middle market strategy is the best, and the performance in the terminal market strategy is the lowest. In conclusion, when the working capital ratio is in different intervals, the performance in the three strategies will appear different phenomena.

### 6.5. Marginal Effect of Working Capital on Performance in the Industry as a Whole

Based on Table 9, we can conclude that the marginal effects of working capital on performance in current strategies are higher than the overall level in the industry as a whole.

**Figure 3:**
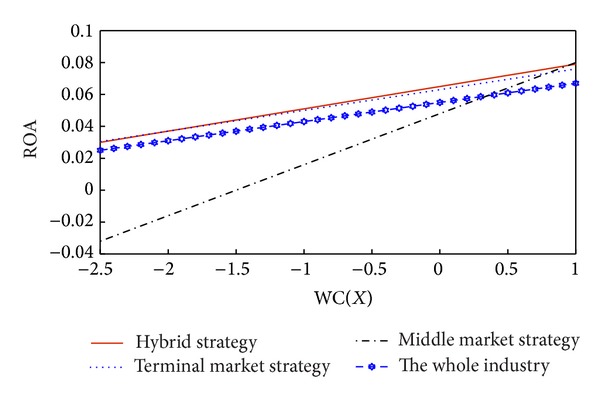
Margin effects of working capital on performance in current strategies and the whole industry.

According to the analysis above, we draw the schematics comprehensively.  Figure 3 shows the marginal effects of working capital on performance in the current strategies and the industry as a whole.

We can see that, when the working capital ratio is above 0.35 in the industry, the performances in the three strategies are higher than the industry level. When the working capital ratio is below 0.35, the performances in the terminal market strategy and the hybrid strategy are higher than the industry level, while the performance in the middle market strategy is lower than the industry level.

According to the above analysis, the results suggest that the strategic choice moderates the effects of the level of working capital on firm performance, and the marginal effects of working capital on performance are different with the different strategies. Furthermore, the strategic choice should fit the level of working capital. The performance would be promoted significantly if the strategic choice is matched with the level of working capital. That is, the company should adjust the level of working capital that corresponds to the different strategic choices to improve firm performance, or enterprises should choose the competitive strategy that best fits with the different levels of working capital to enhance firm performance and competitiveness.

## 7. Conclusion

This paper studies the influence of competitive strategic choice on the working capital configuration and working capital-performance relationship using the wholesale and retail companies listed in the Shenzhen and Shanghai stock market as the research object.

Previously, empirical financial studies ignored the role strategic choices play as a determinant of working capital. The results of the present analysis indicate that the strategic choices developed by the wholesale and retail industry do indeed affect their working capital management. That is, strategic choices will influence management efficiency, the composition, and the adjustment speed of working capital. Strategic choices are clearly a determining factor in working capital management and deserve more attention in future investigations.

Furthermore, with respect to the analysis of working capital determinants (growth opportunities, operational cash flow, management efficiency of working capital, and fixed assets ratio), different strategic choices seem to have different effects on these determinant factors. Another important result of this analysis is that the influence of strategic choices on working capital will finally transfer to performance.

In conclusion, as there is a target working capital ratio and there are limits of transaction cost and size, the working capital ratio we discussed should be in a particular optimal interval. In this interval, the adjustment extent in the hybrid strategy is greater than that in the other two strategies, and its performance is the best and most stable, while the marginal influence of working capital on performance is in the middle. Once a company chooses the hybrid strategy and its working capital policy has been established, the change of working capital will not change its performance excessively because the marginal influence of working capital on performance is not that high. Therefore, companies with this strategy will not pay much attention to working capital. Conversely, companies with the terminal strategy have a lower turnover rate and more cash and short-term borrowings to maintain differentiation, and their working capital adjustment and the marginal influence of working capital on performance is the lowest. Thus they pay little attention to working capital policy. To maintain a better performance, the working capital rate of these companies should be as low as possible. In the middle market strategy, the turnover rate is the largest, and inventories and receivables to current assets are the highest to provide their products to clients as fast as possible. Although the adjustment extent is in the middle, they should also be mindful of working capital because the marginal influence of working capital on performance is the greatest, which can lead their performance to become unstable. Based on the conclusions above, the hybrid strategy is the best strategy choice for companies in terms of performance in a fast developing wholesale and retail industry situated in a relatively fast developing market.

Therefore, while an assessment of working capital management must take into account strategic choices, this conclusion implies that strategic choice is a feature that differentiates between firms on the basis of their financial behaviors. One practical implication of our research is that when managers of wholesale and retail companies make decisions regarding their working capital policy, they should be concerned with the consistency between working capital and their strategic choice because it may influence the working capital adjustment and therefore lead to a different performance. Thus, the effects of strategy on working capital and the coherence between strategy and working capital should receive the utmost attention.

## Figures and Tables

**Table 1:** Variable definitions.

Variable	Definition	
Strategy variables		
Capital investment efficiency		
FTOP	Ratio of fixed assets to profit	((ending net fixed assets_*t*_ + begninning net fixed assets_*t*_)/2)/total profit_*t*_
FTUO	Fixed asset turnover ratio	sales_*t*_/((ending net fixed assets_*t*_ + begninning net fixed assets_*t*_)/2)
CTUO	Current assets turnover ratio	sales_*t*_/((ending current assets_*t*_ + begninning current assets_*t*_)/2)
Cost control		
RME	Ratio of management expenses to profit	administration expenses_*t*_/total profit_*t*_
RFE	Ratio of financial expenses to profit	financial expenses_*t*_/total profit_*t*_
RSE	Ratio of sales expenses to sales	selling expenses_*t*_/sales_*t*_
CTOI	Ratio of cost to income	total cost_*t*_/gross revenue_*t*_
Development capability		
RGM	Relative gross margin	firm's gross profit ratio_*t*_/average of industrial profit ratio_*t*_
GF	Growth rate of fixed assets	(ending net fixed assets_*t*_ − begninning net fixed assets_*t*−1_)/ending net fixed assets_*t*−1_
GSP	Growth rate of sales proceed	(operation revenue_*t*_ − operation revenue_*t*−1_)/operation revenue_*t*−1_
Working capital		
WC	Working capital ratio	(current asset_*t*_ − current liability_*t*_)/current asset_*t*_
Working capital Influencing factors		
Management efficiency of working capital		
ITUO	Inventory turnover ratio	operation costs_*t*_/((ending inventory_*t*_ + begninning inventory_*t*_)/2)
RTUO	Accounts receivable turnover ratio	sales_*t*_/((ending account receivable_*t*_ + begninning account receivable_*t*_)/2)
Growth opportunities		
GSP	Growth rate of sales proceed	(sale_*t*_ − sale_*t*−1_)/sale_*t*_
Operational cash flow		
OCF	Operating cash flow to total assets	operating cash flow_*t*_/total asset_*t*_
Fixed assets ratio		
FA	Fixed assets ratio	fixed asset_*t*_/total asset_*t*_
Firm size		
SIZE	Size of firm	ln⁡(total asset_*t*_)
Performance		
ROA	ROA	EBIT_*t*_/total asset_*t*_
Control variables		
Steady of industry demand		
IDU	Industry demand uncertainty	$\sqrt{\sum_{i=1}^{n}{\left({\text{industrial net sales}}_{i}-\bar{\text{industrial net sales}}\right)}^{2}}$
Initial performance		
ROA	Lag of ROA	EBIT_*t*_/total asset_*t*_
Working capital structure and efficiency		
Working capital efficiency		
ITUO	Inventory turnover ratio	operation costs_*t*_/((ending inventory_*t*_ + begninning inventory_*t*_)/2)
ARTUO	Accounts receivable turnover ratio	sales_*t*_/((ending account receivable_*t*_ + begninning account receivable_*t*_)/2)
Working capital structure		
CA	Cash to current assets	cash_*t*_/current asset_*t*_
RA	Receivables to current assets	ending account receivable_*t*_/current asset_*t*_
IA	Inventories to current assets	ending inventory_*t*_/current asset_*t*_
SL	Short-term loans to current liabilities	ending short term loans_*t*_/current asset_*t*_

**Table 2:** Factor analysis results.

Variables	Factor 1: input-output ability (IOA)	Factor 2: capital investment efficiency (CIE)
FTOP	0.90	−0.03
RFE	0.87	0.03
RSE	0.01	−0.50
RME	0.92	−0.01
FTUO	0.01	0.28
RGM	0.01	−0.99
CTOI	−0.01	0.99
GF	0.01	0.06
GSP	0.004	0.08
CTUO	0.02	0.23
Eigen value	2.44	2.38
Variance contribution	0.49	0.48
Overall contribution	96.54%	
Bartlett test	0.000	
KMO test	0.69	

**Table 3:** Factors in different classes.

Variables	Whole sample		The first class mean	The second class mean	The third class mean	ANOVA on differences between all three clusters (*F*-test and its *P* value are reported)
	Mean	Standard deviation				
IOA	2.44*e* − 09	0.96	0.19	−0.32	0.29	10.87 (0.000)
CIE	−1.02*e* − 08	0.99	−0.03	−1.29	0.38	626.37 (0.000)
WC	−0.23	0.90	−0.17	−0.86	0.38	52.51 (0.000)
Size	21.51	0.89	21.51	21.28	21.67	6.77 (0.0013)
Obs.	475		198	115	162	

**Table 4:** Working capital allocation for the whole sample and comparison across the three strategies.

Variables	Whole sample		Hybrid strategy mean	Terminal market strategy mean	Middle market strategy mean	ANOVA on differences between three clusters (*F*-test and its *P* value )
	Mean	Standard deviation				
Efficiency						
ITUO	18.78	58.13	14.11	13.99	27.90	3.05 (0.0483)
ARTUO	14.95	113.27	3.43	−3.11	41.85	7.24 (0.0008)
Structure						
CA	38.15	21.47	42.40	44.81	28.23	29.97 (0.000)
RA	10.98	12.54	8.27	7.48	16.77	29.54 (0.000)
IA	28.56	18.11	29.05	24.56	30.81	4.18 (0.0159)
SL	30.85	21.09	28.04	37.64	29.46	9.31 (0.0003)
Obs.	475		198	115	162	

**Table 5:** Determinants of working capital in three strategic types.

Variables	Hybrid strategy	Terminal market strategy	Middle market strategy
WC_*t*−1_	0.267^*∗∗∗*^ (0.005)	0.500^*∗∗*^ (0.000)	0.322^*∗∗*^ (0.000)
ITUO_*t*_	−0.555^*∗*^ (0.038)	−2.795 (0.000)	−0.077^*∗∗∗*^ (0.002)
ARTUO_*t*_	−0.076^*∗∗∗*^ (0.000)	−0.380^*∗∗∗*^ (0.010)	−0.361 (0.129)
GSP_*t*_	−0.097^*∗*^ (0.068)	0.012 (0.964)	0.024^*∗∗∗*^ (0.000)
FA_*t*_	−3.288^*∗∗∗*^ (0.000)	−3.774^*∗∗∗*^ (0.000)	−0.232^*∗∗∗*^ (0.007)
OCF_*t*_	0.382 (0.390)	−1.817^*∗∗∗*^ (0.007)	−0.157 (0.178)
Constant	0.876^*∗∗∗*^ (0.000)	1.643^*∗∗∗*^ (0.000)	0.189^*∗∗∗*^ (0.000)
Arellano-Bond test	0.467 (0.641)	−0.527 (0.598)	0.355 (0.723)
Sargan test	10.44 (0.236)	9.70 (0.287)	6.20 (0.625)
Wald test	399.11	1726.35	1220.49

^*∗*^ *P* < 0.05.

^*∗∗*^ *P* < 0.01.

^*∗∗∗*^ *P* < 0.001.

The Arellano-Bond test is a second-order autocorrelation of residuals under the null of no serial correlation.

The Sargan test is a test of the overidentifying restrictions, under the null of instruments' validity.

A Wald test is the significance of estimated coefficients.

**Table 6:** Measures of interaction of margin influence.

IO × WC	IO × WC	Variables	
*≠*0	=0	IO × WC_*i*,*t*_^(1)^	Working capital ratio in hybrid strategy
=0	*≠*0	IS × WC_*i*,*t*_^(2)^	Working capital ratio in terminal market strategy
=0	=0		Working capital ratio in middle market strategy

**Table 7:** Distribution of working capital ratio.

Distribution	[−1,1]	[−2, −1]	[−3, −2]	[−4, −3]	[−5, −4]	<−5

Obs.	421	33	10	1	9	1

**Table 8:** Influence of working capital on performance in different strategies.

Variables	Model 1	Model 2
WC_*i*,*t*_	0.012^*∗∗*^ (0.011)	0.032^*∗∗∗*^ (0.004)
IO_*i*,*t*_		0.017^*∗∗*^ (0.013)
IS_*i*,*t*_		0.015^*∗*^ (0.054)
IO × WC_*i*,*t*_^(1)^		−0.018 (0.136)
IS × WC_*i*,*t*_^(2)^		−0.019^*∗*^ (0.098)
IDU	−0.001^*∗∗*^ (0.011)	−0.001^*∗∗∗*^ (0.009)
ROA_*i*,*t*−1_	0.440^*∗∗∗*^ (0.000)	0.421^*∗∗∗*^ (0.000)
Constant	0.055^*∗∗∗*^ (0.000)	0.048^*∗∗∗*^ (0.000)
Wald Test	142.86	142.61
*R*-square	0.765	0.733

^∗^ *P* < 0.05.

^∗∗^ *P* < 0.01.

^∗∗∗^ *P* < 0.001.

ROA_*i*,*t*−1_ is explained by the one period lag for ROA.

**Table 9:** Margin effects of working capital on performance in different strategies and in the whole industry.

	The whole industry	Hybrid strategy	Terminal market strategy	Middle market strategy
Margin	0.012	0.014	0.013	0.032
Intercept	0.055	0.065	0.063	0.048

**Table 10:** Performance in the different strategies when the working capital ratio is in different intervals.

	[−2, −1.63]	[−1.63, 0.75]	[0.75, 0.95]	[0.95,1]
Terminal market strategy	Highest	Middle	Lowest	Lowest
Middle market strategy	Lowest	Lowest	Middle	Highest
Hybrid strategy	Middle	Highest	Highest	Middle

## References

[1] Smith KV (1973). State of the art of working capital management. *Financial Management*.

[2] Frecka TJ, Lee CE (1983). Generalized financial ratio adjustment processes and their implications. *Accounting Research*.

[3] Hamlin AP, Heathfield DF (1991). Competitive management and working capital. *Management and Decision Economics*.

[4] Groth JC (1992). The operating cycle: risk, return and opportunities. *Management Decision*.

[5] David JS, Hwang Y, Pei BKW, Reneau JH (2002). The performance effects of congruence between product competitive strategies and purchasing management design. *Management Science*.

[6] Deloof M (2003). Does working capital management affect profitability of Belgian firms?. *Journal of Business Finance and Accounting*.

[7] Raheman A, Afza T, Qayyum A, Bodla MA (2010). Working capital management and corporate performance of manufacturing sector in Pakistan. *International Research Journal of Finance and Economics*.

[8] Kim YH, Chung KH (1990). An integrated evaluation of investment in inventory and credit: a cash flow approach. *Journal of Business Finance & Accounting*.

[9] Wang Y (2002). Liquidity management, operating performance, and corporate value: evidence from Japan and Taiwan. *Journal of Multinational Financial Management*.

[10] Faulkender M, Wang R (2006). Corporate financial policy and the value of cash. *Journal of Finance*.

[11] Kieschnick R, LaPlante M, Moussawi R (2011). Working capital management and shareholder wealth. *Working paper*.

[12] Ding S, Guariglia A, Knight J (2013). Investment and financing constraints in China: does working capital management make a difference?. *Journal of Banking & Finance*.

[13] Shin HH, Soenen L (1998). Efficiency of working capital management and corporate profitability. *Financial Practice and Education*.

[14] Appuhami B (2008). The impact of firms capital expenditure on working capital management an empirical study across industries in Thailand. *International Management Review*.

[15] Khan GA, Ghazi IU (2013). Working capital management and firm performance in Karachi Stock Exchange (KSE). *Management and Administrative Sciences Review*.

[16] Baños-Caballero S, García-Teruel PJ, Martínez-Solano P (2012). How does working capital management affect the profitability of Spanish SMEs?. *Small Business Economics*.

[17] Baños-Caballero S, García-Teruel PJ, Martínez-Solano P (2014). Working capital management, corporate performance, and financial constraints. *Journal of Business Research*.

[18] Forte M, Hoffman JJ, Lamont BT, Brockmann EN (2000). Organizational form and environment: an analysis of between-form and within-form responses to environmental change. *Strategic Management Journal*.

[19] Luo Y, Park SH (2001). Strategic alignment and performance of market-seeking minces in China. *Strategic Management Journal*.

[20] Valos MJ, Bednall DHB, Callaghan B (2007). The impact of Porter’s strategy types on the role of market research and customer relationship management. *Marketing Intelligence & Planning*.

[21] Hofmann E (2010). Linking corporate strategy and supply chain management. *International Journal of Physical Distribution and Logistics Management*.

[22] Wenelfelt B (1984). A resource-based view of the firm. *Strategic Management Journal*.

[23] Barney JB (1991). Firm resources and sustained competitive advantage. * Journal of Management*.

[24] Grant RM (1991). The resource-based theory of competitive advantage: implications for strategy formulation. *California Management Review*.

[25] Peteraf MA (1993). The cornerstones of competitive advantage: a resource-based view. *Strategic Management Journal*.

[26] Collis DJ, Montgomery CA (1995). Competing on resources: strategy in the 1990s. *Harvard Business Review*.

[27] Oliver C (1997). Sustainable competitive advantage: combining institutional and resource-based views. *Strategic Management Journal*.

[28] Greene PG, Brown TE (1997). Resource needs and the dynamic capitalism typology. *Journal of Business Venturing*.

[29] Nath P, Nachiappan S, Ramanathan R (2010). The impact of marketing capability, operations capability and diversification strategy on performance: a resource-based view. *Industrial Marketing Management*.

[30] Conant JS, Mokwa MP, Varadarajan PR (1990). Strategic types, distinctive marketing competencies and organizational performance: a multiple measures-based study. *Strategic Management Journal*.

[31] Hill CWL, Hitt MA, Hoskisson RE (1992). Cooperative versus competitive structures in related and unrelated diversified firms. *Organization Science*.

[32] Woodside AG, Sullivan DP, Trappey RJ (1999). Assessing relationships among strategic types, distinctive marketing competencies, and organizational performance. *Journal of Business Research*.

[33] La Rocca M, La Rocca T, Gerace D, Smark C (2009). Effect of diversification on capital structure. *Accounting & Finance*.

[34] Baños-Caballero S, García-Teruel PJ, Martínez-Solano P (2010). Working capital management in SMEs. *Accounting and Finance*.

[35] Porter M (1980). *Competitive Strategy*.

[36] Kotha S, Nair A (1995). Strategy and environment as determinants of performance: evidence from the Japanese machine tool industry. *Strategic Management Journal*.

[37] Kobrin SJ (1991). An empirical analysis of the determinants of global integration. *Strategic Management Journal*.

[38] Sanches R (1995). Strategic flexibility in product competition. *Strategic Management Journal*.

[39] Kim C, Mauer DC, Sherman AE (1998). The determinants of corporate liquidity: theory and evidence. *The Journal of Financial and Quantitative Analysis*.

[40] Opler T, Pinkowitz L, Stulz R, Williamson R (1999). The determinants and implications of corporate cash holdings. *Journal of Financial Economics*.

[41] Denis DJ, Sibilkov V (2010). Financial constraints, investment, and the value of cash holdings. *Review of Financial Studies*.

[42] Brown JR, Petersen BC (2011). Cash holdings and R&D smoothing. *Journal of Corporate Finance*.

[43] Denis DJ (2011). Financial flexibility and corporate liquidity. *Journal of Corporate Finance*.

[44] Tong Z (2011). Firm diversification and the value of corporate cash holdings. *Journal of Corporate Finance*.

[45] Bhagat S, Bolton B (2008). Corporate governance and firm performance. *Journal of Corporate Finance*.

[46] Mintzberg H (1978). Patterns in strategy formation. *Management Science*.

[47] Reger RK, Huff A (1993). Strategic groups: a cognitive perspective. *Strategic Management Journal*.

[48] Ozkan A (2001). Determinants of capital structure and adjustment to long run target: Evidence from UK company panel data. *Journal of Business Finance and Accounting*.

[49] Miles RE, Snow CC (1994). *Fit, Failure and the Hall of Fame*.

[50] Ginsberg A, Venkatraman N (1985). Contingency perspectives of organizational strategy: a critical review of the empirical research. *Academy of Management Review*.

[51] Aragón-Correa JA, Sharma S (2003). A contingent resource-based view of proactive corporate environmental strategy. *Academy of Management Review*.

